# Possible Role of Leptin in Atopic Dermatitis: A Literature Review

**DOI:** 10.3390/biom11111642

**Published:** 2021-11-05

**Authors:** Carlos Jiménez-Cortegana, Germán Ortiz-García, Amalia Serrano, David Moreno-Ramírez, Víctor Sánchez-Margalet

**Affiliations:** 1 Department of Medical Biochemistry and Molecular Biology and Immunology, School of Medicine, Virgen Macarena University Hospital, University of Seville, 41009 Seville, Spain; cjcortegana@gmail.com (C.J.-C.); gerortgar@alum.us.es (G.O.-G.); 2 Department of Medicine, School of Medicine, Dermatology Service, Virgen Macarena University Hospital, 41009 Seville, Spain; amaliaserranog@gmail.com (A.S.); dmoreno@us.es (D.M.-R.)

**Keywords:** atopic dermatitis, obesity, inflammation, leptin, adipokine, immune system

## Abstract

Atopic dermatitis (AD) is the most frequent chronic inflammatory skin disease, and its incidence has been rapidly increasing in developed countries in the last years. AD presents a high degree of heterogeneity due to biases and confounding factors such as age range, sex, or ethnicity. For those reasons, the search for new biomarkers is crucial. At the same time, obesity, which is a global health problem, has also increased over the years. It has been associated with many pathophysiological states, including skin diseases such as AD, mostly in childhood. Obesity promotes a low grade inflammation driven by many different cytokines and adipokines, including leptin, which has a key role in many other diseases due to its pleiotropic effects. Leptin also has a role in both skin and allergic diseases very related to AD. Thus, this adipokine could have an important role in the pathogenesis of AD, especially in its chronicity. Despite the limited literature available, there is some evidence that leads us to consider leptin as an important adipokine in this skin disease. For this reason, here we have reviewed the role of leptin in the pathophysiology of AD.

## 1. Introduction

AD is one of the most common chronic skin diseases, and its prevalence has mainly increased in developed countries, affecting around 15–20% of children and 1–3% of adults [1]. AD aetiology is complex, since it involves genetic components, alterations of the skin barrier function, immune system dysfunction and environmental factors [2]. Thus, AD is a highly heterogeneous disease in which there are differences between intrinsic vs. extrinsic phenotypes [3], children vs. adults [4], or between racial or ethnic groups [5]. Moreover, AD patients usually have personal or family history of asthma, allergic rhinitis, food allergies or urticaria, and the quality of life is usually impaired due to the intense itching that this condition bears [6].

AD aetiology is also affected by obesity, which has been demonstrated to produce a pro-inflammatory condition that locally and systemically affects AD [7]. Obesity is considered as the major public health problem since its prevalence has undergone a dramatic increase in recent decades, especially in developed countries [8], causing many comorbidities due to its relationship with multiple pathologies such as autoimmune [9] or inflammatory diseases [10]. In obese individuals, this is led by an inflammatory response that seems to be supported by tissue impairment or homeostatic imbalance, in which adipokines, soluble proteins secreted by the white adipose tissue (WAT), act as mediators [11].

Currently, there are no reliable biomarkers to distinguish AD from other skin diseases [12] and its search remains challenging to support their clinical use. On the one hand, AD severity is categorised into mild, moderate, or severe, but AD is a heterogeneous disease and there are no specific biomarkers to differentiate between severity groups. In this sense, one study concluded that the combination of four serum biomarkers, which are thymus and activation-regulated chemokine (TARC)/CC chemokine ligand 17 (CCL17), pulmonary and activation-regulated chemokine (PARC)/CCL18, interleukin (IL)-22, and sIL-2R, were better correlated with AD severity than individual biomarkers [13], although TARC/CCL17 alone may also be a good marker [14]. In other cases, AD severity has been correlated with serum levels of other markers but without reliable results, including CD30, Macrophage-Derived Chemoattractant (MDC), IL-12, IL-16, IL-18, and IL-31, and TARC [12]. Esaki et al. (2016) concluded that the acute phase of AD was associated with a strong Th2/Th22 activation with the contribution of Th17 cells, whereas the chronic phase was characterised by Th1 polarisation, although the Th2 pathway could still play an important role in this phase. As a Th2 disease, AD pathophysiology also involves type 2 innate lymphoid cells (ILC2), mast cells, basophils and eosinophils [2]. In addition, more Th17-related cytokines and antimicrobial peptides were detectable in the skin of AD children compared with their adult counterparts. Th9/IL-9 and IL-33 were increased in pediatric AD skin [15,16]. IL-31 has also been found in AD children compared with AD adults, but without differences in thymic stromal lymphopoietin serum concentrations between age groups [17].

On the other hand, AD can be divided into extrinsic and intrinsic groups depending on the presence or lack of immunoglobulin (Ig) E. This Ig cannot be considered as a good biomarker either because some patients have shown to develop high IgE levels throughout the course of the disease [18] and elevated allergen-specific IgE levels have also been found in healthy individuals [19]. Moreover, AD severity has not been correlated with IgE levels in some cases [18,20]. In addition, cell populations such as eosinophils [21] and mast cells [22] have been evaluated without consistent results and high levels of Th2 cytokines have been found in the skin lesions of both AD phenotypes, although some studies have suggested that Th2 polarisation is predominant in extrinsic AD [16]. Other studies suggested that Th17 and Th22 cells were elevated in AD, especially in the intrinsic phenotype [23].

Due to the absence of both consistent and reliable biomarkers, obesity-induced inflammation could play a key role in AD. One of the most important pro-inflammatory obesity-related adipokines is leptin, which has pleiotropic functions [24]. Moreover, *overweight* and *obesity* were associated with an increased risk of this disorder, thus suggesting a relationship between leptin and skin diseases such as atopic dermatitis (AD) [25]. For this reason, the purpose of this article is to review the available literature related to the possible role of this adipokine in AD, and to take leptin into account as a possible biomarker in the disease.

## 2. Leptin at a Glance

Obesity promotes a low-grade, chronic inflammation mediated by the secretion of hormones and cytokines, including leptin [25], which has been demonstrated to stimulate the production of pro-inflammatory cytokines in immune cells [26] as well as inducing reactive oxygen species [27]. Leptin is a soluble peptide hormone consisting of 167 amino acids whose existence was predicted for the first time in the 1950s and 1960s on leptin deficient (ob/ob) and leptin receptor deficient (db/db) mice [28,29], although it was finally described in 1994 as the product of the *obese* (Ob or LEP) *gene* [30], with a structure similar to the long-chain helical cytokine family, which includes interleukin (IL) 6, IL-11, IL-12, G-CSF or oncostatin M, among others [31]. Leptin is produced not only in WAT, a dynamic organ with multiple functions (e.g., the regulation of metabolism and energy balance, or its involvement in both inflammatory and immune responses), but also other tissues such as placenta [32], mammary gland [33], ovary [34] or pituitary gland [35]. Leptin plays a key role in the regulation of both energy metabolism and obesity, and also takes part in different diseases and processes, including (but not limited to) reproduction [36], autoimmunity [37], glucose metabolism [38], non-alcoholic fatty liver disease [39] or cancer [40,41].

**Figure 1:**
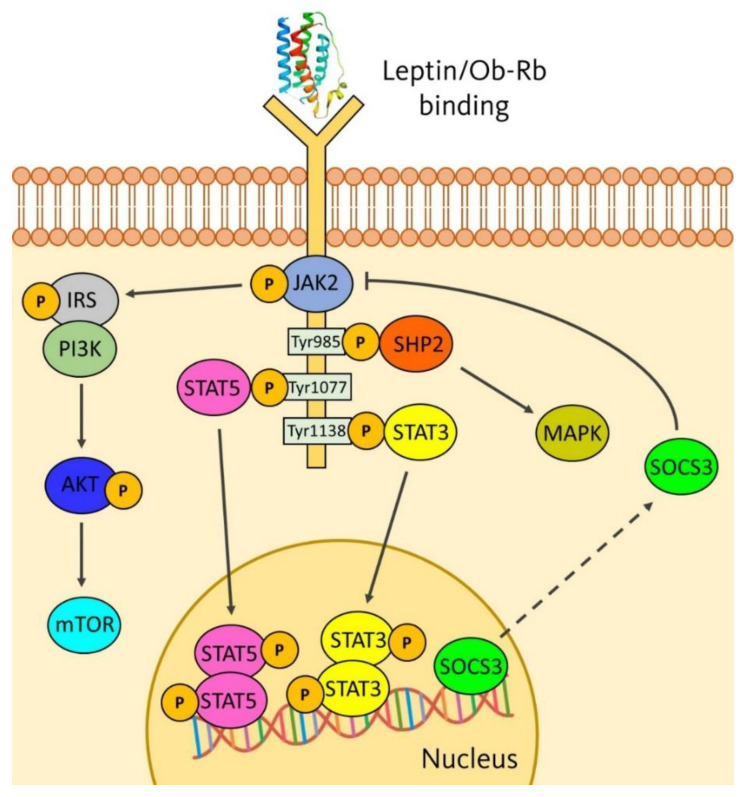
Mechanism of leptin action and activation of signalling pathways.

Leptin needs to bind their receptors (Ob-Rs) to carry out its functions. Ob-R belong to the long-chain helical cytokine family [42] and are widely distributed throughout the organism [43]. There is a huge variety of leptin receptors, called Ob-Ra, Ob-Rb, Ob-Rc, Ob-Rd, Ob-Re and Ob-Rf, which confer leptin its pleiotropic effects by activating signalling pathways, such as Janus kinase (JAK) 2/signal transducer and activator of transcription (STAT) 3, insulin receptor substrate (IRS)/phosphatidylinositol-3 kinase (PI3K), among others. Intracellular signalling pathways are illustrated in Figure 1. They are first activated via JAK2 phosphorylation. Then, three tyrosine residues, located in the intracellular domain of Ob-Rb, are phosphorylated (Tyr985, Tyr1077 and Tyr1138) [44,45]. Tyr985 induces the SHP2 signalling pathway and the activation of ERK1/2 of the MAPK family [46], Tyr1077 residue mediates the transcriptional activation of STAT5 gene [47], and Tyr1138 mediates the activation of STAT3. This gene is dimerised and translocated to the nucleus to activate transcription of target genes such as the suppressor of cytokine signalling (SOCS)-3 gene, a negative feedback of leptin signalling [46], whose overexpression causes resistance not only to leptin but also to insulin [48]. JAK2 also modulates the phosphorylation of the IRS family (IRS1 and IRS2) and, consequently, activates the PI3K/Akt/mTOR pathway, which is crucial in many aspects related to cell growth and survival, such as cancer [49].

## 3. Role of Obesity in AD

Obesity is considered as a risk factor due to its relationship with insulin resistance, carcinogenesis, and cardiovascular, musculoskeletal or brain diseases [50,51], as well as skin diseases [52]. Obesity has been demonstrated to have a strong association with AD in children [53], increasing the risk of developing AD as soon as they become obese [54]. By contrast, both positive and negative associations in adults between obesity and AD have been reviewed [55]. The stronger relationship in childhood could be due to the immaturity of the immune system at these ages, when hypersensitivity reactions are more likely to occur, with obesity increasing the immune imbalance [54]. In adults, contradictory results could have been obtained, in part, due to confounding factors (e.g., age groups, race, educational levels, industrialisation, urban life, or family income), which interfere in analyses and make significant associations in some regions and not in others [7,51,55].

There are many factors involved in the connection between obesity and AD. For example, obesity alters the skin function by causing lower permeability to evaporate water loss compared with normal weight controls [56]. Obesity could promote many skin diseases such as acrochordons or keratosis pilaris, which are produced by circulatory and lymphatic changes [57], and may develop malignant melanoma or non-melanoma skin cancer [58], although this relationship is controversial [59]. This is due to the role of obesity causing a systemic state, increasing the release of both pro-inflammatory cytokines and adipokines (e.g., TNF-α, IL-6, and leptin) [25]. Moreover, AD seems to be more frequent in women [60], as they tend to have a higher degree of body fat and more subcutaneous WAT in both the abdominal and gluteofemoral area than men with similar levels of total adiposity and BMI [61]. Women also have a metabolically active adipose tissue because aromatase transforms androgens into estrogens. In addition, 17-β-estradiol levels are increased in obese women, which could influence the relationship between AD and obesity [62]. Estrogens can also regulate eosinophil migration and survival, and the secretion of IL-3 and IL-4 by monocytes, leading a T helper type (Th) 2 response, thus being able to favour a state of chronic inflammation [62,63].

Other studies have suggested the possible relationship between obesity and the microbiome in AD. Brandwein et al. (2019) demonstrated strong associations between BMI and skin microbiome, in which *Corynebacterium* abundance was highly correlated [64]. Another study showed that both dysbiosis in *Faecalibacterium prausnitzii* and dysregulation of gut epithelial inflammation could affect the progression of AD [65]. In AD children, the gut microbiota has shown to be characterised by a dysbiotic status with a prevalence of some microbes such as *Faecalibacterium, Oscillospira, Bacteroides, Parabacteroides* and *Sutterella*, and the reduction or lack of others with inflammatory effects, such as *Bifidobacterium, Blautia*, *Coprococcus*, *Eubacterium* and *Propionibacterium* [66]. Moreover, Son et al. (2019) performed a protocol for a case-control study to better understand the association between obesity and AD in terms of gut microbiome, metabolome, and immunological mechanisms to improve future management strategies for AD [67].

Adipokines different from leptin have also been investigated in AD. Adiponectin and resistin have been found to be inversely correlated to the intensity of eczema in adult patients compared with healthy subjects, thus being considered as promising biomarkers in the disease [68,69]. Specifically, the resistin rs3745367 gene polymorphism could contribute to the development of AD according to gender and age [69,70]. Surprisingly, Machura et al. (2013) found higher levels of serum resistin in AD children (especially in boys) compared with healthy controls. Serum apelin levels were also higher, especially in girls, and visfatin was significantly lower compared with their healthy counterparts [71].

## 4. Role of Leptin in AD

The first aspects related to serum leptin and AD began to be studied two decades ago. Up to now, only twelve studies have investigated leptin in AD (Table 1). In some cases, the association between leptin and AD was controversial and some studies had no clinical significance, mainly due to the difficulty in separating this pathology from biases and confounding factors, such as age groups, sex, race, educational levels, industrialisation, urban life, or family income [7,51,55].

High leptin levels were found by Kimata (2002,2006) in children with IgE-associated AD, especially in those with fatty liver [72,75]. Another study also showed high leptin in AD children, mainly in girls [81]. In addition, teenagers, and adults with AD, both with normal and high BMI had high leptin concentrations compared with controls [83]. Jaworek et al. (2020) reported similar results, but leptin was not correlated with AD severity, sex, age or BMI [68]. By contrast, low leptin levels were significantly found in lactating adult women with AD compared with their healthy counterparts [81], as well as in AD children, in which leptin was inversely associated with the severity of AD [79]. Nonetheless, other studies did not show a relationship between this skin disease and leptin in neither children [74] nor teenagers and adults [77]. However, in the latter case mentioned, patients with allergic contact dermatitis had higher levels of leptin compared with both AD patients and controls [77]. The severity of AD and leptin has also been found to be uncorrelated in subjects at any age [76,80].

Another confounding factor that could interfere with the results is AD phenotype. AD has been classically distinguished into extrinsic and intrinsic types, clinically identical entities but differing each other in the concentration of IgE, which is higher in the extrinsic form [10,83]. In this sense, some of the twelve studies did not find associations between leptin and IgE in both teenagers and adults [68,82], which suggests that the role of leptin in AD pathogenesis may be due to other inflammatory mechanisms not mediated by IgE, or reported a negative trend in children, thus suggesting that leptin is associated with intrinsic AD [81]. By contrast, obesity has been positively correlated with high levels of both leptin and IgE, aggravating AD in rats [78]. Han et al. (2016) found positive correlations between obesity and leptin, and obesity and IgE, but they did not directly measure the relationship between leptin and IgE in their cohort [80]. Another study found a significant IgE level in AD children but its association with leptin was not analysed [74].

Another factor involved in leptin measures could be corticosteroids, which are the first-line treatment for AD [84]. Systemic corticosteroid has been shown to significantly increase serum leptin [85], but topical steroid did not influence leptin levels [74]. Conversely, not only has leptin concentration been investigated but also polymorphisms of the leptin gene. A Jordanian study analysed the association of three leptin gene polymorphisms (rs7799039, rs791620, and rs2167270) with the incidence of AD in 164 patients compared with 167 controls. Interestingly, only rs2167270 polymorphism was significantly correlated with AD, and GG allele was more prevalent in patients (38.7%) than in controls (26.1%) [86].

**Figure 2:**
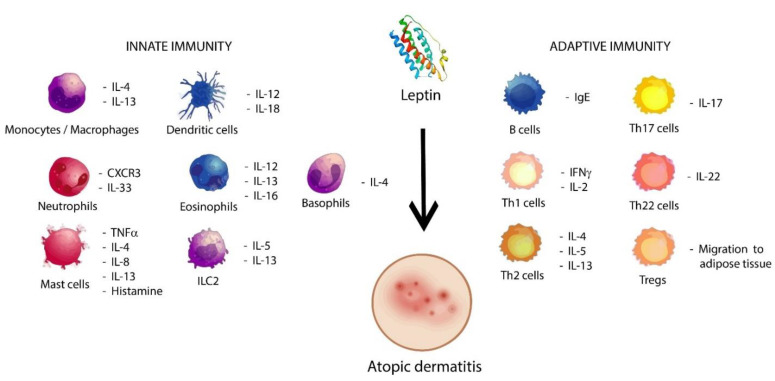
Probable role of leptin in atopic dermatitis. Leptin induces the activation of cells from both the innate and adaptive immunology system, which are able to promote the activation and proliferation of cytokines and other cells in pro-allergic conditions, such as Th2 immune responses, ILC2, and type 2 cytokines, thus contributing to atopic dermatitis.

All those different results reflect the heterogeneity of AD, in which immune dysfunctions are also involved [87], as shown in Figure 2. AD is considered as a T cell-mediated disease [88] that mostly involves Th1, Th2, and regulatory T cells (Tregs) [87]. In this sense, leptin has two major mechanisms of action in the immune response. The first is the regulation of the Th1/Th2 balance through cytokine production, which has been studied in obese asthmatics [89] and to promote Th1/Th17 cell survival via ERK1/2 and AKT-mTOR signalling pathways [90], thus improving the production of cytokines such as IFN-γ or TNF-β, which have been found to be upregulated in the blood signature of AD patients [91]. The second mechanism consists of the downregulation of Tregs driven by leptin and its receptors [92], causing a loss of inhibitory signalling in those cells, which is associated with chronic infections [44]. However, Tregs have been found highly expanded in AD [93,94], probably because adipose tissue, which is characterised by high serum leptin levels, was demonstrated to be a preferent location for accumulation of Tregs, commonly known as adipose tissue-resident Tregs [95,96]. This suggests that Treg measures may be another confounding factor in the link between leptin and AD depending on their location. On the other hand, current data also suggest that susceptibility to cutaneous infections are mainly produced by abnormalities in the innate system [97] and leptin has a key role in the immunomodulation of type 2 responses, which characterise AD [2]. In this sense, STAT3, MAPK, and mTORC1 pathways are also activated by leptin to enhance Th2 responses [98], the predominant form in AD [99], which release IL-4, IL-5 or IL-13, and increase the production of IgE, thus aggravating skin inflammation and skin barrier defects in AD [100]. Moreover, eosinophilia has been found in this skin disease [101] and leptin can promote eosinophil migration and activation [102]. Pérez-Pérez et al. (2017) reviewed that leptin could stimulate the chemotaxis of eosinophils, the release of inflammatory cytokines such as IL-1β and IL-6, and both the activation and inhibition of apoptosis in those cells, thus considering Ob-R as a survival factor in eosinophils [103]. In line with this, positive associations have been found between eosinophilic activity with serum leptin and TNF-α levels in atopic asthmatic obese children and adolescents [104], which suggest that leptin could play an immunopathophysiological key role in allergic inflammation of obese patients with asthma [105]. Other Th2 immune responses in which leptin is involved are basophil migration and survival [106], the pro-inflammatory activity of mast cells [107] and IL-17 production via ILC2 in allergic rhinitis [108].

## 5. Overview and Concluding Remarks

Leptin has been suggested as a promising biomarker in some diseases previously mentioned in the introductory section and it also may play an important role in AD for some reasons. First, this adipokine was correlated with Th1 polarisation in obese children with asthma [90] and Th1 responses could take part in the chronic phase of AD [109]. Since leptin promotes the differentiation towards Th1 immune responses, obese patients could be more susceptible to chronic AD. In the same line of this hypothesis, an American study treated AD patients with phototherapy and most of the overexpressed genes and inflammatory cells were decreased. However, some genes did not respond properly, including leptin. The expression of this adipokine decreased in the epidermis and increased in the dermis, without returning to normal levels after treatment [110]. This suggests that high leptin expression in the dermis may promote immune activation and chronicity of AD, which could mainly affect obese patients. Furthermore, leptin has also shown important effects on type 2 innate immune cells, including eosinophils, basophils, mast cells and ILC2, which have been demonstrated to be critical for the development of AD.

Although there is evidence concerning the role of both obesity and leptin in many diseases and in the immune system (e.g., leptin promotes other skin and allergic diseases [25,111]), we should take into account the high degree of heterogeneity of AD, which could affect leptin analyses. This review has allowed us to find out that there are conflicting and controversial data for leptin in this skin disease, mainly due to both the small cohort size analysed in some studies and the classification of AD in different groups (severity, phenotype, age, race, or gender) as well as other parameters including (but not limited to) the type of corticosteroid used, IgE levels, BMI, or cytokine production.

In conclusion, it is clear that further experimental pre-clinical and clinical research is needed to better identify the role of leptin in AD and its probable use as a clinical circulating biomarker. In addition, since obesity seems to have a promising role in AD microbiota, a new research avenue on the role of leptin in the microbiota of AD patients may be considered in future studies.

## Figures and Tables

**Table 1:** Existing studies related to the role of leptin in atopic dermatitis (AD) and its possible relationship with IgE in the disease. NM: not measured (correlation analysis was not performed). No correlation: correlation analysis was performed but without statistically significant differences.

Study (Year)	Country	Type of Study	Life Stage	Subjects *	Correlation Leptin-AD	Correlation Leptin-IgE
Jaworek et al., (2020) [68]	Poland	Humans	Adults	79	Positive	No correlation
Kimata (2002) [72]	Japan	Humans	Children	50	Positive	Positive
Kimata (2004) [73]	Japan	Humans	Adults	60	Negative	NM
Bostanci et al., (2004) [74]	Turkey	Humans	Children	40	No correlation	NM
Kimata (2006) [75]	Japan	Humans	Children	1226 **	NM	NM
Nagel et al., (2009) [76]	Germany	Humans	Children	462	NM	NM
Balato et al., (2011) [77]	Italy	Humans	Teenagers and adults	138	No correlation	NM
Jeong et al., (2015) [78]	Korea	Rats	Pups	33	Positive	NM
Seo et al., (2016) [79]	Korea	Humans	Children	227	Negative	NM
Han et al., (2016) [80]	Korea	Humans	Children, teenagers, and adults	64	No correlation	NM
Mohammed et al., (2017) [81]	Egypt	Humans	Children	90	Positive	Negative
Jung et al., (2020) [82]	Korea	Humans	Teenagers and adults	40	Positive	No correlation

* Include sample sizes for both AD and comparison groups. ** This study comprised a total of 1226 individuals distributed in smaller groups between 1999 and 2003.

## Data Availability

Not applicable.
